# Advancing Parkinson’s Prevention: Brain-Specific Lipopolyplex Delivery of the GDNF as a Neuroprotective Gene Therapy

**DOI:** 10.1021/acsmaterialsau.6c00024

**Published:** 2026-03-24

**Authors:** Yujing Zhang, Yujing Huang, Zhonghua Lu, Zhen Yuan

**Affiliations:** † Ministry of Education, Frontiers Science Center for Precision Oncology, Faculty of Health Sciences, 59193 University of Macau, Macau, SAR 999078, China; ‡ Center for Cognitive and Brain Sciences, Institute of Collaborative Innovation, University of Macau, Macau, SAR 999078, China; § Research Center for Primate Neuromodulation and Neuroimaging, Institute of Biomedical and Health Engineering, Shenzhen Institute of Advanced Technology, 85411 Chinese Academy of Sciences, Shenzhen 518055, China; ∥ Shenzhen Technological Research Center for Primate Translational Medicine, Shenzhen Key Laboratory for Molecular Biology of Neural Development, Shenzhen-Hong Kong Institute of Brain Science, Shenzhen Institute of Advanced Technology, Chinese Academy of Sciences, Shenzhen 518055, China

**Keywords:** Parkinson’s disease, gene therapy, glial cell line-derived neurotrophic factor, lipopolyplex, brain targeting

## Abstract

Parkinson’s disease (PD) is characterized by progressive dopaminergic neuron loss. Although the glial cell line-derived neurotrophic factor (GDNF) offers therapeutic promise, its clinical translation is hampered by challenges related to delivery methods and the timing of intervention. Here, we developed a brain-targeted lipopolyplex (BAGLPP) for systemic GDNF gene delivery. BAGLPP incorporates an RVG29 peptide-modified lipid shell for blood–brain barrier crossing and a polyethylenimine-condensed AAV plasmid core for efficient transfection. Following intravenous administration, BAGLPP demonstrated superior brain accumulation and expression in PD-relevant regions. In a cellular PD model, BAGLPP pretreatment reduced apoptosis, oxidative stress, and pathological α-synuclein (pS129) accumulation. In MPTP-induced mice, prophylactic BAGLPP treatment established sustained GDNF expression (maintaining levels 3.7-fold higher than controls at 8 weeks postlesion), which activated the PI3K/Akt pro-survival pathway, suppressed pro-apoptotic Bax, and attenuated neuroinflammation. This multifaceted protection preserved dopaminergic neurons and striatal dopamine and improved motor function without significant systemic toxicity. Our findings establish BAGLPP as a durable, nonviral gene therapy platform with strong potential for preventing PD progression.

## Graphical abstract

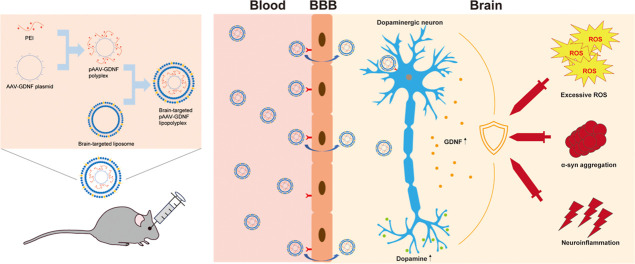

## 1 Introduction

Parkinson’s disease (PD), acknowledged as the second most prevalent neurodegenerative disorder, affects more than ten million individuals worldwide with a projected doubling of cases by 2040.^1^ Pathologically, PD is defined by the progressive degeneration of dopaminergic neurons within the substantia nigra pars compacta (SNpc) alongside striatal dopamine depletion, leading to motor symptoms such as tremor, rigidity, and bradykinesia.^2^ Current treatment, including dopamine replacement therapy,^3–5^ lifestyle modifications,^6,7^ and surgery,^8–10^ focuses on symptom management rather than cure. Given that the symptoms of PD become manifested only after the loss of at least half of the dopaminergic neurons in the substantia nigra, strategies aimed at neuronal protection and regeneration offer promising therapeutic potential.

While the precise etiology of PD remains incompletely understood, factors such as excessive accumulation of α-synuclein, elevated levels of reactive oxygen species (ROS), and neuroinflammation contribute to disease progression.^2^ Enhancing neuronal resilience to these stressors may help prevent or slow PD. The glial cell line-derived neurotrophic factor (GDNF) has demonstrated a relatively high selectivity for dopaminergic neurons in PD and has shown potential in safeguarding midbrain neurons from degeneration.^11–13^ While viral vector-based GDNF gene therapy has demonstrated sustained neuroprotection in preclinical models,^14,15^ limitations such as pre-existing immunity, insertional mutagenesis risks, and inability to redose hinder clinical translation.^16^ Despite ongoing efforts, clinical translation of GDNF therapy has been hindered primarily by challenges related to delivery methods and the timing of intervention.^17,18^ Thus, developing safe and efficient methods for the early cerebral delivery of the GDNF is crucial.

Nonviral vectors offer safer and scalable alternatives, but their applications are hindered by low transfection efficiency and poor targeting.^19^ Among these, lipopolyplexes (LPPs)—hybrid nanoparticles combining liposomes and polymers (e.g., polyethylenimine, PEI)—exhibit superior nucleic acid encapsulation, endosomal escape, and elevated biocompatibility.^20,21^ Further modification with targeting ligands enables precise brain delivery.^22,23^ The 29-amino acid rabies virus glycoprotein peptide (RVG29) has been shown to facilitate blood—brain barrier translocation and neuron-specific targeting^24,25^ via binding to nicotinic acetylcholine receptors (nAChRs)^26,27^ or γ-aminobutyric acid-type B (GABA_B_) receptor.^28^

To synergize the benefits of viral and nonviral systems, we engineered RVG29-functionalized LPPs to deliver AAV-derived plasmids encoding the GDNF (pAAV-GDNF) driven by a robust constitutive cytomegalovirus early enhancer/chicken β-actin (CAG) promoter. This hybrid design leveraged the high transfection efficiency of AAVs’ inverted terminal repeat (ITR)-flanked transgene cassette while avoiding viral particle limitations.^29^ Unlike conventional AAVs, which require complex capsid engineering for redosing, LPPs enable repeated plasmid delivery without triggering neutralizing antibodies. In this study, we developed a brain-targeting LPP to deliver pAAV-GDNF (designated as BAGLPP) for the prevention of PD in a 1-methyl-4-phenyl-1,2,3,6-*tetra*-hydropyridine (MPTP)-induced mouse model. BAGLPP construction involved a PEI-condensed pAAV-GDNF core coated with RVG29-modified lipids. We optimized component ratios for high transfection efficiency and demonstrated successful brain targeting and transgene expression in the striatum and substantia nigra following intravenous administration.

In vitro and in vivo studies revealed that BAGLPP reduced α-synuclein aggregation, oxidative stress, and neuroinflammation, thereby attenuating neuronal loss and restoring dopamine levels. Moreover, BAGLPP improved motor function in PD mice without significant adverse effects. We further investigated its capacity for sustained GDNF expression and elucidated the underlying prosurvival and antiapoptotic mechanisms. This work aims to provide a translatable strategy for durable neurotrophic support to halt or slow the PD progression.

## 2 Methods and Materials

### 2.1 Materials and Reagents

25 kDa linear polyethylenimine (PEI) was purchased from Beyotime Biotechnology. Dipalmitoyl-phosphatidylcholine (DPPC), cholesterol, 1-methyl-4-phenylpyridinium (MPP^+^) iodide, and 1-methyl-4-phenyl-1,2,3,6-tetrahydropyridine (MPTP) hydrochloride were purchased from Sigma-Aldrich. DSPE-PEG2000 and DSPE-PEG2000-RVG29 were purchased from MedChemExpress and Xi’an Ruixi Biological Technology, respectively.

### 2.2 Cell Culture and Animals

SH-SY5Y human neuroblastoma cells were cultivated in Dulbecco’s modified Eagle’s medium (DMEM) supplemented with 10% fetal bovine serum (FBS) and 1% penicillin/streptomycin under standard conditions (37 °C, 5% CO_2_ in a humid atmosphere).

Eight-week-old male C57BL/6J mice (about 25 g) were provided by the Animal Research Core at the Faculty of Health Science, University of Macau, and kept under controlled temperature and humidity with free access to food and water in the animal facility. All animal procedures were approved by the University of Macau Animal Ethics Committee (UMARE-021-2023).

### 2.3 Preparation of the Lipopolyplex

#### 2.3.1 Preparation of Plasmids

The plasmid pAAV-CAG-EYFP was acquired from Addgene (#104055). The GDNF expression plasmid (pAAV-CAG-GDNF) was synthesized by Genwiz via replacement of the EYFP sequence with the mouse GDNF coding sequence. An empty vector served as a control. Plasmids were amplified by cloning them into the DH5α strain of *E. coli* and purified by using Tiangen EndoFree Midi-Prep kits (TIANGEN) following the manufacturer’s instructions. The plasmids were checked by Nanodrop (ThermoFisher) to assess purity and concentration before the experimental test.

#### 2.3.2 Preparation of Polyplexes

The PEI/DNA polyplex (PP) was prepared by mixing PEI and the plasmid through electrostatic interactions between the positively charged nitrogen in PEI and the negatively charged phosphate in DNA. In a typical polyplex preparation, 1 μg of plasmid was dissolved in 50 μL of complexation buffer (150 mM NaCl, 10 mM HEPES, pH 7.4). In addition, different amounts of PEI (1 mg/mL solution) were dissolved in 50 μL of the same buffer (mPEI/mDNA = 1 denotes that 1 μg of plasmid was mixed with 1 μg of PEI). The PEI solution was then pipetted into the plasmid solution and immediately vortexed. Subsequently, the mixture was incubated for 30 min at room temperature. The final DNA concentration was 10 ng/μL.

#### 2.3.3 Preparation of Liposomes

Liposomes were prepared by using a thin-film hydration method.^30^ The ratios of DPPC to DSPE-PEG2000-(RVG29) and to cholesterol were optimized to balance the stability and transfection efficiency, based on a previous report.^20,31^ A lipid mixture of DPPC, cholesterol, and DSPE-PEG2000 (or DSPE-PEG2000-RVG29) in chloroform/methanol (2:1, v/v) was dried under N_2_, vacuum-desiccated, and hydrated with ddH_2_O to 5 mg lipid/mL. Then the liposome was extruded 11 times through a 100 nm polycarbonate membrane by using a heated Mini-Extruder (Avanti Polar Lipids) with a 1000 μL Hamilton syringe (Hamilton). Lipid content was quantified colorimetrically.

#### 2.3.4 Preparation of Lipopolyplexes

An appropriate amount of liposomes (from Section 2.3.3) was diluted in 50 μL of DEPC H_2_O, mixed with 50 μL of the PEI/DNA polyplex suspension containing 1 μg of DNA (from 2.3.2) by vigorously pipetting up and down, and then incubated at room temperature for 60 min to obtain the lipopolyplex (LPP). After sterilization via a 0.22 μm filter membrane (Beyotime, Shanghai, China), the LPPs were stored at 4 °C.

### 2.4 Characterizations of Polyplexes, Liposomes, and Lipopolyplexes

The hydrodynamic size and zeta potential of each complex were determined by dynamic light scattering using a ZetaSizer Nano-ZS instrument (Malvern Instruments Ltd., Malvern, UK). For hydrodynamic size, 50 μL of the complex was diluted in 2 mL of ddH_2_O in a glass cuvette (DTS0012), with each program set for 10 runs and each complex repeated 3 times. Then the diluted solution was transferred to a folded capillary cell (DTS1070) to test the zeta potential. Samples were measured at 25 °C. The morphology of BAGLPP was observed by transmission electron microscopy after being negatively stained with uranyl acetate from Beijing Zhongkebaice Technology Service Co., Ltd. (www.zkbaice.cn). BAGLPP was incubated with 30% FBS at 37 °C for different time periods to test serum stability.

### 2.5 Agarose Gel Electrophoresis

An appropriate amount of agarose powder (Bio-Rad) was dissolved in 1 × TAE buffer (Beyotime) after heating in a microwave oven to obtain a 1% agarose gel mixture. The DNA sample was mixed with DNA loading buffer (ThermoFisher) containing GelRed dye (Sigma) and run in a TAE buffer at 100 V for 20 min. The bands were then visualized under a ChemiDocTM Touch Imaging System (Bio-Rad, USA).

### 2.6 Cell Transfection

SH-SY5Y cells were plated at 4 × 10^4^ cells/well in 24-well plates (Corning). After 24 h in culture, PPs or LPPs were added at amounts corresponding to 0.5 μg of DNA each well. Besides, 0.5 μg of DNA transfected by lipo3000 reagent (ThermoFisher) following the manufacturer’s instructions was used as the control. The medium was replaced after 6 h. After 48 h of transfection, cells were washed and analyzed by flow cytometry to determine transfection efficiency. The transfection images were detected in 24-well plates under an Axiovert 40 CFL fluorescence microscope.

### 2.7 Determination of the Cellular Uptake Mechanism

SH-SY5Y cells were plated at 4 × 10^4^ cells/well in 24-well plates and adhered for 24 h. Then, cells were pretreated for 1 h at 37 °C with various endocytosis inhibitors dissolved in a serum-free medium: 10 μM chlorpromazine (MCE) to inhibit clathrin-mediated endocytosis, 5 mM methyl-β-cyclodextrin (MCE) to disrupt caveolae-mediated endocytosis by depleting cholesterol, and 100 μM genistein (MCE) to inhibit autophagy-lysosome endocytosis.^32^ BAELPP was transfected into each well, and the transfection efficiency was determined by flow cytometry after 48 h of transfection as described in Section 2.6.

### 2.8 MPP^+^-Induced In Vitro PD Model and Treatment Groups

To establish the in vitro PD model, SH-SY5Y cells were seeded in plates and allowed to adhere for 24 h. Subsequently, the culture medium was replaced with a fresh medium containing 1 mM MPP^+^. Cells were exposed to MPP^+^ for a duration of 24 h to induce cytotoxicity. To access the neuroprotection of BAGLPP, SH-SY5Y cells were transfected with BAGLPP for 6 h and the medium was replaced with a fresh complete medium. Following a 42 h gene expression period post-transfection, cells were then treated with 1 mM MPP^+^ for 24 h. Specific assays were performed immediately after the MPP^+^ treatment period, unless stated otherwise.

### 2.9 Cell Viability Assay

Cell viability was analyzed by using Cell Counting Kit 8 (CCK8, Beyotime) according to the manufacturer’s protocols. To assess the toxicity of PP or LPP, cells were seeded and cultured at a density of 5 × 10^3^/well in 100 μL of the medium into 96-well microplates (Corning). And then cells were treated as indicated in the cell transfection section at amounts corresponding to 0.1 μg of DNA each well. After treatment for 24 h, 100 μL of fresh DMEM with 10% CCK-8 reagent was added to each well and then cultured at 37 °C for 1.5 h. To assess the neuroprotection of BAGLPP, after cells were treated with MPP^+^ for 24 h, 100 μL of fresh DMEM with 10% CCK-8 reagent was added to each well and then cultured for 1.5 h. The absorbance at 450 nm was measured with a microplate reader (Bio-Rad). The data were normalized to the results of the PBS control group. All experiments were performed in triplicate.

### 2.10 ROS Detection Assay

2,7-Dichloro-dihydro-fluorescein diacetate (DCFH-DA) (Beyotime) was diluted by 1:1000 in a fresh medium and added to treated SH-SY5Y cells cultured in 24-well plates, 200 μL per well. After incubation for 20 min at 37 °C, the cells were harvested and washed three times with PBS. The percentage of DCFH-DA-positive SH-SY5Y cells was detected by a CytoFLEX FLOW Cytometer (Beckman Coulter). The mean fluorescence intensity of DCFH-DA was detected in a confocal dish under an Axiovert 40 CFL fluorescence microscope (Zeiss) and analyzed by ZEN 3.2 software.

### 2.11 Apoptosis Detection

An Annexin V-FITC Apoptosis Detection Kit (Beyotime) was used to detect cell apoptosis. The cells were harvested and resuspended in 195 μL of binding buffer in a 1.5 mL centrifuge tube. Then, 5 μL of Annexin V-FITC and 10 μL of propidium iodide (PI) were added to the tube and incubated at room temperature away from light for 30 min with slow shaking. Next, all samples were placed on ice and examined by flow cytometry.

### 2.12 Flow Cytometry

A CytoFLEX FLOW Cytometer (Beckman Coulter) was used to perform flow cytometry in this study. For DNA transfection and ROS detection, cells were harvested and resuspended in phosphate-buffered saline (PBS) (Gibco). For each sample, a minimum of 10,000 events were collected within the live cell gate, which was defined based on forward scatter (FSC) and side scatter (SSC) parameters. Flow cytometry was performed in the FITC channel to analyze EYFP expression and DCFH-DA fluorescence, and cells whose fluorescence intensities exceeded the maximum of the untransfected control group were considered positive cells. Transfection efficiency was calculated as the percentage of EYFP-positive cells within the live cell population. For cell apoptosis detection, flow cytometry was performed in the FITC and PE channels. All acquisition speeds were set at low (10 μL/min), and each treatment was repeated three times. FlowJo V10 software was used to analyze the flow cytometry results.

### 2.13 Western Blotting

For electrophoresis, a standard vertical twin plate mini gel unit (Bio-Rad) was used. The protein sample was obtained by RIPA lysate (Beyotime), and concentrations of the proteins were determined by a BCA protein assay kit (Beyotime). Three volumes of samples were mixed with one volume of 4× Laemmli sample buffer (Bio-Rad) with 10% 2-mercaptoethanol (Sigma, MO, USA). Samples were loaded in a 4%–20% BeyoGel SDS-PAGE Precast Gel (Beyotime) and performed at a constant voltage of 100 V for 40 min in 1× running buffer (Bio-Rad). Then the gel was semidry-transferred to polyvinylidene fluoride (PVDF) membrane (Bio-Rad) at a constant current of 1.0 A for 7 min in 1× semidry-transferred buffer (Bio-Rad). The membranes were blocked in 3% bovine serum albumin (BSA) in PBST (PBS containing 0.05% Tween 20) for 1 h at room temperature (RT). Primary antibodies were diluted in primary antibody dilution buffer (Beyotime) and incubated overnight at 4 °C. The membranes were washed 3 times in PBST and incubated with secondary antibodies (diluted in 3% BSA in PBST) for 2 h at RT. The membranes were washed 3 times in PBST and visualized with Clarity Western ECL Substrate (Biorad) under a ChemiDoc Touch Imaging System (Bio-Rad). The corresponding antibody is listed in Supporting Information Table 2. ImageJ software was used to analyze the optical density of the bands.

### 2.14 In Vivo Lipopolyplex Delivery through Retro-orbital Injection

In the in vivo study, the preparation of LPPs was previously detailed in Section 2.3, highlighting increased quantities of the plasmid, PEI, and liposomes. The concentration of DNA utilized in the LPP was standardized at 100 ng/μL. Freshly prepared LPP was administered via retro-orbital injection into C57BL/6J mice in a volume of 100 μL over a period of seven consecutive days. Anesthesia was induced with 3% isoflurane. Post-induction, gentle pressure was applied to the skin to protrude in the right eye. A 1 mL insulin syringe, with the bevel oriented away from the eye, was carefully inserted into the retrobulbar sinus, where LPPs were slowly injected.

### 2.15 In Vivo and Ex Vivo Imaging

Three weeks postinjection, the mice were anaesthetized and imaged using an AniView Phoenix in vivo imaging system (BLT photon technology) with a 500/580 nm filter set. Then the mice were sacrificed, and their brains and other main organs were extracted for ex vivo imaging. The mean fluorescence intensity in the region of interest (ROI) was recorded as photons per second per centimeter squared per steradian (p/s/cm^2^/sr) and exported from AniView Phoenix software for further analysis.

### 2.16 Immunofluorescence Staining and Analysis

Three weeks post-LPP injection, the mice were anesthetized with an overdose of 2.5% Avertin solution (2,2,2-tribromoethanol in 2-methyl-2-butanol, Sigma) and perfused through the heart with phosphate-buffered saline (PBS) (Gibco) to remove all blood and postfixed in 4% paraformaldehyde (PFA) (Solarbio) at 4 °C overnight. The brains were then dehydrated by 30% sucrose in PBS and further frozen and embedded by optimal cutting temperature compound (OCT) (ThermoFisher) and sliced into 50 μm coronal sections in a Leica CM3050S frozen section machine (Leica). The brain slices were stored in an antifreeze solution (PBS: glycol: glycerol = 5:3:2) at −20 °C in a refrigerator until the assay. Brain slices were washed three times in PBS for 10 min each. Blocking was performed for 1 h in 0.3% Triton X-100 + 5% BSA in PBS. Then, the primary antibody was diluted in 0.3% Triton X-100 + 5% BSA in PBS and incubated in a dark environment at 4 °C for one night. After washing three times in PBS, the samples were incubated with the secondary antibody diluted in 0.3% Triton X-100 + 5% BSA in PBS and incubated for 2 h at room temperature, followed by washing four times in PBS. DAPI (Beyotime) was added to the samples, incubated for 15 min at room temperature, and then washed. Further, high-resolution imaging was performed by a Zeiss LSM 710 confocal microscope. The relative fluorescence intensity was analyzed by using ZEN 3.2 software. To quantify the survival of dopaminergic neurons, regions of interest (ROIs) were first identified. The number of fluorescent protein and DAPI double-positive cells within each ROI was manually counted using Photoshop in a single-blind manner and averaged across 5 consecutive coronal sections. The corresponding antibody is listed in Supporting Information Table 2.

### 2.17 MPTP-Induced In Vivo PD Model

A PD mouse model induced by MPTP was established according to a previous protocol.^33^ A fresh MPTP-HCl solution diluted in physiological saline was prepared on the day of injection and then injected intraperitoneally daily for 5 consecutive days at a dose of 30 mg/kg. Likewise, the normal control group was injected with an equal volume of saline in the same way.

### 2.18 Open-Field Test

Open-field testing was carried out to assess the locomotor behavior of mice for different treatment groups. Before the tests, the mice were placed in the testing room and handled by the experimenter for 3 days for adaptation. A camera was used to measure movements of the test animals and was vertically placed on a 50 × 50 × 50 cm polyvinyl chloride box, and it recorded 5 min after the mice were placed in the bottom center in each trial. The open field was wiped with ethanol solution and dried between each test to remove the odor trails. The total traveled distance and mobile/immobile time were recorded and analyzed in SMART V3.0 software (RWD).

### 2.19 Rotarod Test

Mice were trained on a Rotarod (Xinruan) at a speed of 10 rpm for 3 days before MPTP treatments. During the test, the speed of the Rotarod accelerated from 5 to 40 rpm, and the holding time of each mouse was recorded.

### 2.20 Preparation of Brain Tissue Homogenates

Mice were sacrificed, and brain tissues were extracted in ice, in which the corresponding regions of the brain were separated. For Western blot analysis, the tissue was stirred in RIPA lysis buffer with a protease inhibitor (Beyotime), and the supernatant was then collected by centrifuging at 12,000*g* for 20 min. For the enzyme-linked immunosorbent assay (ELISA), the tissues were stirred in PBS with a protease inhibitor, and the supernatant was collected. The protein concentration was tested with the BCA kit.

### 2.21 Enzyme-Linked Immunosorbent Assay

Cell culture supernatants at different time points after transfection were collected, and the protein concentrations were measured by a BCA protein assay kit (Beyotime). The GDNF level was detected by a Mouse Glial Cell Line Derived Neurotrophic Factor (GDNF) ELISA Kit (JL10491, Jianglai) according to the manufacturer’s protocol. Expressions of dopamine (D751019, Sangon Biotech), tumor necrosis factor-α (TNF-α) (EMC102a, Neobioscience), and interleukin-6 (IL-6) (EMC004, Neobioscience) were measured with corresponding ELISA kits.

### 2.22 Blood Biochemistry and Blood Routine Examinations

Blood was collected in 1.5 mL centrifuge tubes via eye socket bleeding after the mice had been anesthetized with an overdose of 2.5% Avertin solution. 200 μL of blood was transferred to an anticoagulation tube for a routine blood test. For blood biochemistry examination, the whole blood was centrifuged at 12,000*g* for 20 min to collect serum for analysis. Blood biochemistry and routine blood tests were conducted by Servicebio Technology.

### 2.23 Statistical Analysis

Data were presented as the mean ± standard error of the mean (SEM). For comparisons between two groups, statistical significance was determined using an unpaired, two-tailed Student’s *t*-test. For comparisons among three or more groups, one-way analysis of variance (ANOVA) was applied, followed by Tukey’s post hoc test for multiple comparisons. A *p*-value of less than 0.05 was considered statistically significant. The post hoc power analysis was determined by G*Power 3.1.9.7 software to ensure a statistical power >0.8. All statistical analyses were performed using GraphPad Prism software (Version 9.0, GraphPad Software Inc., USA).

## 3 Results

### 3.1 Optimization of Lipopolyplex Composition

The lipopolyplex (LPP) was assembled from three core components: liposomes, the cationic polymer PEI, and plasmid DNA (Figure 1A). We first constructed two AAV plasmids, pAAV-GDNF and pAAV-EYFP, driven by the CAG promoter. To optimize the formulation, we systematically evaluated the PEI/DNA mass ratio (mPEI/mDNA). PEI can absorb the plasmid through electrostatic interaction between the positively charged nitrogen in PEI and the negatively charged phosphate in DNA. Agarose gel electrophoresis confirmed complete DNA condensation at mPEI/mDNA ≥1 (Figure 1B). The transfection efficiency in SH-SY5Y cells increased with the PEI ratio up to 6, establishing mPEI/mDNA = 6 as optimal (Figure 1C). Concurrently, a liposome (DPPC: cholesterol = 85:15) exhibiting a superior transfection performance was constructed based on the present study.^31^ Additionally, the stoichiometry of LPP was evaluated by varying the amounts of liposomes added. Increasing this ratio from 0.2 to 250 progressively reduced the zeta potential from +13.9 ± 0.8 mV to −5.8 ± 0.1 mV and decreased the particle size from 567.6 ± 94.9 to 175.1 ± 3.0 nm at a ratio of 5 (Figure 1D). The transfection efficiency was maximal at a liposome/PEI mass ratio of 5 (Figure 1E). Consequently, a final mass ratio of liposome/PEI/DNA = 30:6:1 was adopted for all subsequent LPP preparations, balancing a high gene delivery efficiency with favorable physicochemical properties.

**Figure 1:**
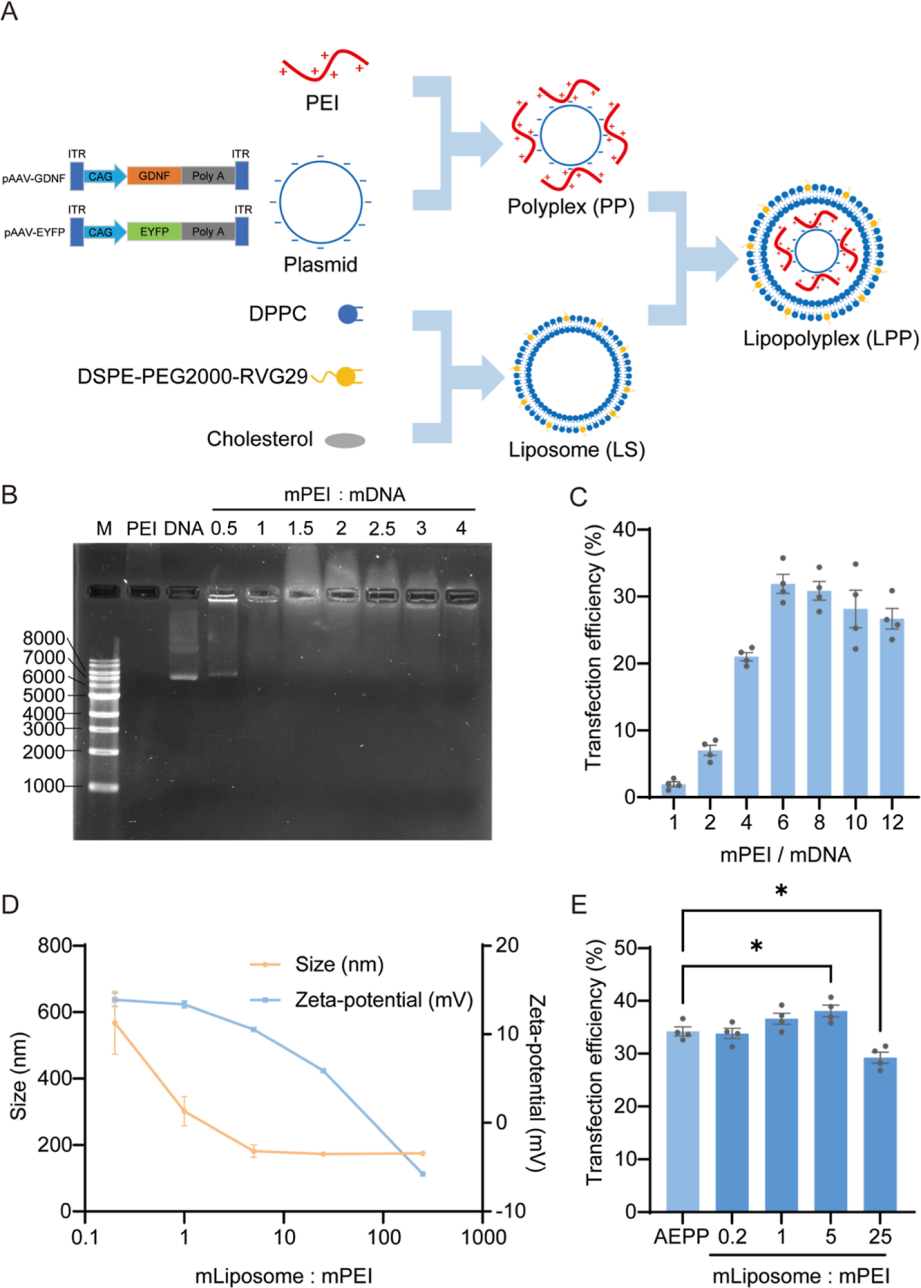
Preparation and characterization of the lipopolyplex (LPP). (A) Schematic illustration of the LPP structure and assembly. (B) Gel electrophoresis analysis of PEI/DNA polyplexes at different mass ratios (mPEI/mDNA). M, DNA marker; the plasmid band corresponds to 6554 bp. (C) Transfection efficiency of PEI/pAAV-EYFP polyplexes in SH-SY5Y cells, expressed as the percentage of EYFP-positive cells (*n* = 4). (D) Hydrodynamic size and zeta potential of LPPs formulated with varying liposome-to-PEI mass ratios (*n* = 3). (E) Transfection efficiency of LPPs with different liposome-to-PEI ratios, compared with the PEI/DNA polyplex (AEPP) (*n* = 4). Data are presented as mean ± SEM; *p < 0.05.

### 3.2 Characterization and Biocompatibility of Lipopolyplexes

According to previous studies, a 10 mol % PEGylation in LPP can enhance blood circulation and reduce uptake by the liver and spleen.^20^ Consequently, we incorporated 10 mol % DSPE-PEG2000-RVG29 to develop a brain-targeting lipopolyplex. A schematic illustration of the self-assembly process of LPP is shown in Figure 1A. Initially, we constructed brain-targeting liposome (BLS) (DPPC:DSPE-PEG2000-RVG29:cholesterol = 85:10:15) and control liposomes (LS) (DPPC:DSPE-PEG2000:cholesterol = 85:10:15), respectively. Subsequently, PEI/pAAV-EYFP (AEPP) was coated with LS to create the pAAV-EYFP lipopolyplex (AELPP), while AEPP and PEI/pAAV-GDNF (AGPP) were coated with BLS to generate brain-targeting AELPP (BAELPP) and brain-targeting AGPP (BAGLPP), respectively. Dynamic light scattering (DLS) revealed sizes of 165.7 ± 0.7 nm (LS), 165.4 ± 3.8 nm (AELPP), 208.1 ± 6.3 nm (BLS), 186.9 ± 3.9 nm (BAELPP), and 185.5 ± 2.0 nm (BAGLPP) (Figure 2A). Transmission electron microscopy (TEM) confirmed the spherical core–shell morphology of BAGLPP (Figure 2A). Coating the anionic liposomes with cationic PPs increased the zeta potential by approximately +12 mV (Figure 2B). Detailed information regarding the size and zeta potential of the compound is provided in Supporting Information Table 1. Similar to the previous research, the PEI polyplex complex exhibited large aggregates (size >1000 nm) and a high polydispersity index (PDI).^31,34^ The gel retardation assay demonstrated efficient condensation and protection of AAV plasmids by the PPs and subsequent LPPs (Supporting Information Figure 1). Furthermore, BAGLPP protected the plasmid payload from serum nuclease degradation for up to 4 h (Supporting Information Figure 2). Importantly, BAELPP showed significantly reduced cytotoxicity compared with the PEI polyplex alone (AEPP) (Figure 2C). The transfection efficiencies of AELPP and BAELPP were comparable to that of AEPP, outperforming the pAAV-EYFP lipoplex formulation (AELS) using the commercial lipo3000 reagent (Figure 2D,F). Next, to investigate the internalization pathway of BAELPP, we employed specific pharmacological inhibitors of different endocytic routes. As shown in Figure 2E, pretreatment with chlorpromazine (CPZ), a potent inhibitor of clathrin-mediated endocytosis, caused a greater than 50% reduction in the cellular uptake of BAELPP. In contrast, inhibition of caveolae-mediated endocytosis by methyl-β-cyclodextrin (M-βCD) and autophagy-lysosome endocytosis by genistein had no significant effect. These results strongly indicated that the cellular entry of BAELPP into SH-SY5Y cells is predominantly mediated by clathrin-dependent receptor-mediated endocytosis. Hence, the optimized LPP could serve as a safe and highly effective gene delivery vector.

**Figure 2:**
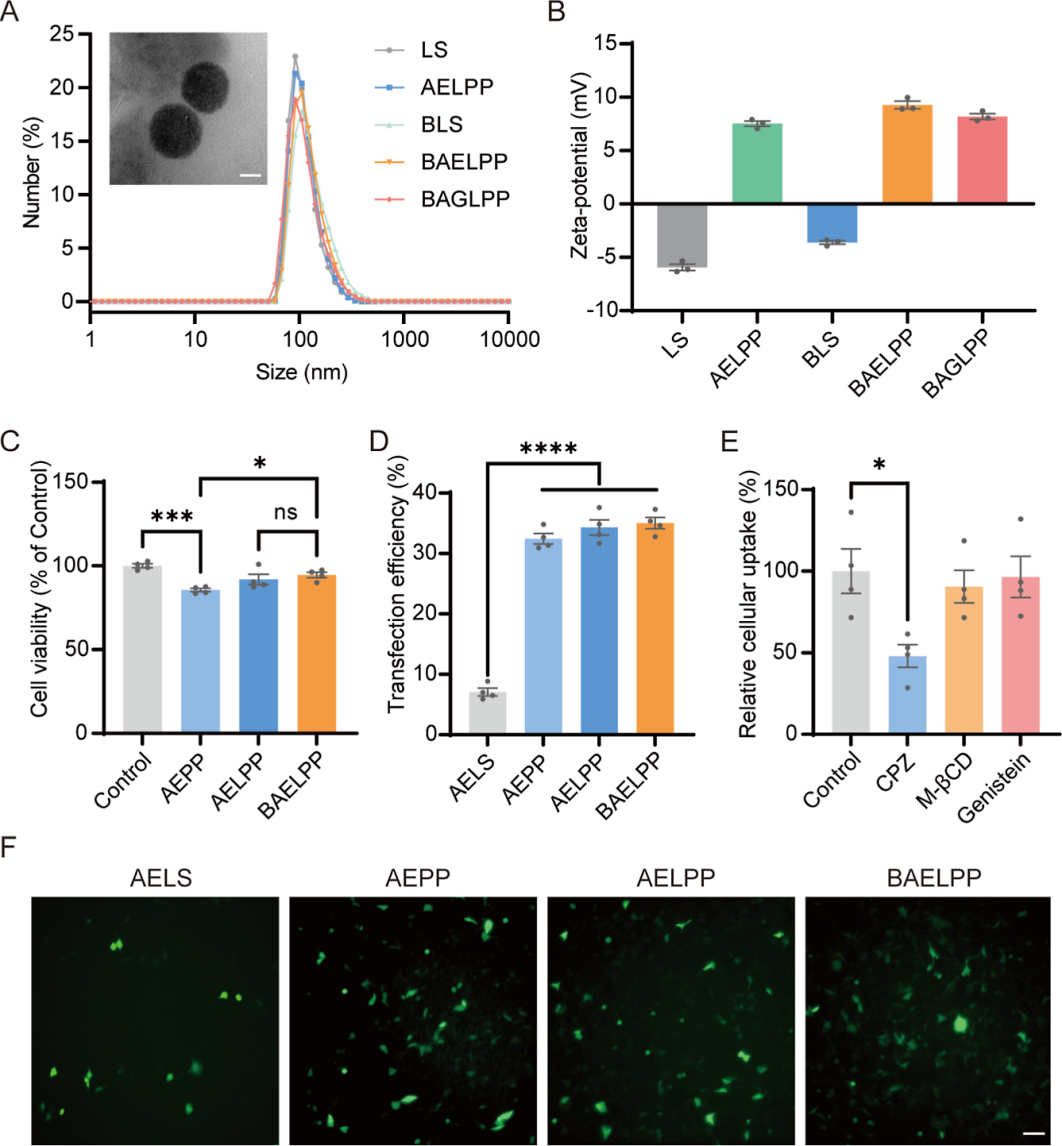
Characterization and functional evaluation of lipopolyplexes (LPPs). (A) Hydrodynamic size distribution of LS, AELPP, BLS, BAELPP, and BAGLPP in ddH_2_O; representative TEM image of BAGLPP (scale bar: 50 nm). (B) Zeta potential of the indicated formulations in ddH_2_O (*n* = 3). (C) Cytotoxicity of AEPP, AELPP, and BAELPP relative to medium control (*n* = 4). (D) Transfection efficiency (% EYFP-positive cells) of AELS, AEPP, AELPP, and BAELPP in SH-SY5Y cells (*n* = 4). (E) Effect of endocytosis inhibitors—chlorpromazine (CPZ), methyl-β-cyclodextrin (M-βCD), and genistein—on BAELPP transfection efficiency (*n* = 4). (F) Representative fluorescence images of EYFP expression after transfection with indicated formulations (scale bar: 100 μm). Data are presented as mean ± SEM; *p < 0.05, ***p < 0.001, ****p < 0.0001; ns, not significant.

### 3.3 Enhanced Brain Targeting of the Lipopolyplex Mediated by RVG29

The BBB poses a significant challenge to the delivery of brain-targeting therapeutics, thereby limiting the effectiveness of interventions for PD. We next evaluated the brain-targeting capability conferred by RVG29 in vivo. Mice received retro-orbital injections of BAELPP (RVG29+) or AELPP (RVG29−). In vivo and ex vivo imaging at 3 weeks postinjection demonstrated significantly stronger EYFP fluorescence signals in the brains of BAELPP-treated mice, with minimal off-target organ signal (Figure 3A–E). Immunofluorescence analysis of brain sections further confirmed EYFP expression in PD-relevant regions—the striatum and substantia nigra—of the BAELPP group (Figure 3F,G). These results validate RVG29 as an effective ligand for enhancing the brain delivery of LPPs.

**Figure 3:**
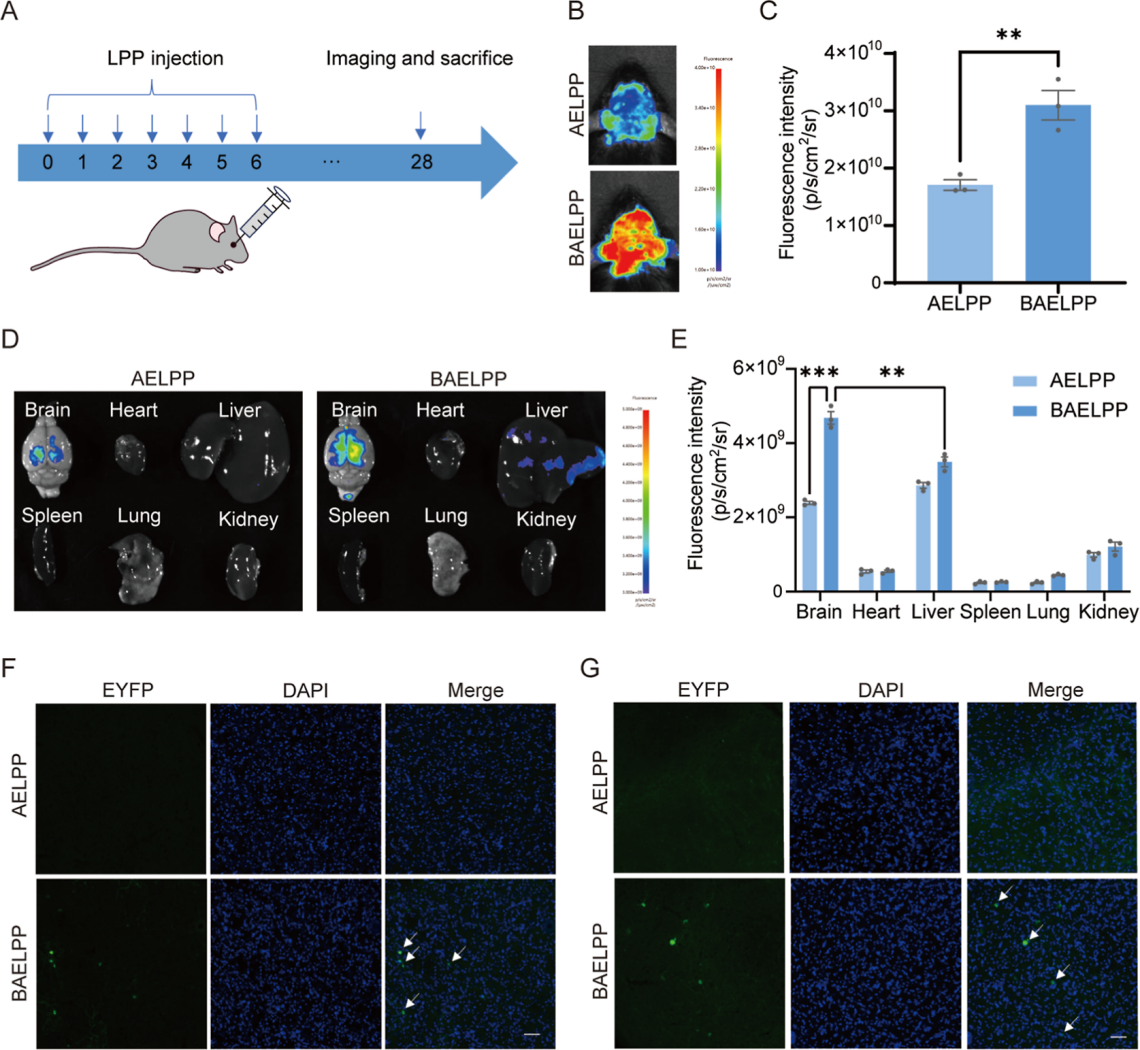
Brain targeting of BAELPP in healthy mice. (A) Schematic of the experimental timeline for retro-orbital injection and imaging. (B) Representative in vivo fluorescence images in the brain of AELPP and BAELPP. (C) Quantification of in vivo brain fluorescence intensity (*n* = 3). (D) Ex vivo fluorescence images of major organs after administration of AELPP or BAELPP. (E) Quantification of ex vivo organ fluorescence intensity (*n* = 3). (F,G) Representative immunofluorescence images of EYFP expression (green) in the striatum (F) and substantia nigra pars compacta (G); nuclei are stained with DAPI (blue). White arrows indicate EYFP-positive cells. Scale bar: 50 μm. Data are presented as mean ± SEM; **p < 0.01, ***p < 0.001.

### 3.4 Neuroprotective Effect of BAGLPP on MPP^+^-Induced SH-SY5Y Cells

The neuroprotective effects of BAGLPP were investigated by utilizing SH-SY5Y cells exposed to methyl-4-phenylpyridinium (MPP^+^). MPP^+^ iodide is widely applied as a model drug for PD to induce Parkinsonian-like alterations in SH-SY5Y nerve cells in vitro.^35^ BAGLPP treatment led to time-dependent GDNF secretion into the supernatant over 84 h (Figure 4A). Because the green fluorescence produced by the transfection of BAELPP has an influence on ROS or apoptosis detection, we constructed a control LPP containing an empty AAV vector (BALPP). Pretreatment with BAGLPP for 48 h prior to MPP^+^ exposure significantly preserved cell viability compared to BALPP (Figure 4B) and reduced the apoptosis rate from 31.32 ± 1.12% to 11.80 ± 0.84% detected by Annexin V-FITC/PI staining (Figure 4C)

**Figure 4:**
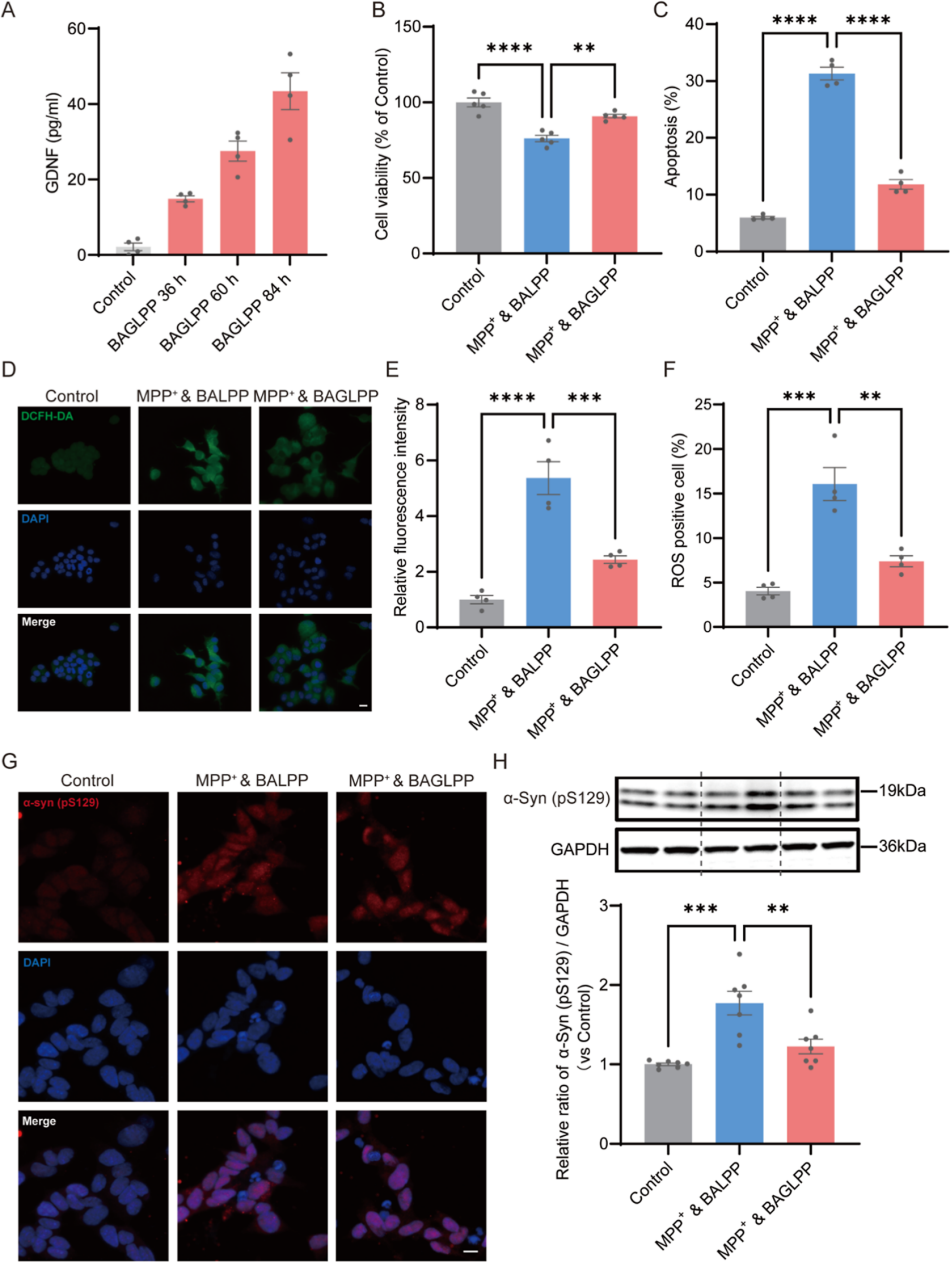
BAGLPP confers neuroprotection in MPP^+^-treated SH-SY5Y cells. (A) Time course of GDNF expression in SH-SY5Y cells after BAGLPP transfection (*n* = 4). (B) Cell viability following pretreatment with BALPP or BAGLPP prior to MPP^+^ exposure (*n* = 5). (C) Apoptosis rates (early + late apoptotic cells) across treatment groups (*n* = 4). (D) Representative images of DCFH-DA (green, ROS) and DAPI (blue, nuclei) staining. Scale bar: 10 μm. (E) Quantification of DCFH-DA fluorescence intensity (*n* = 4). (F) Percentage of ROS-positive cells in each group (*n* = 4). (G) Representative immunofluorescence of phosphorylated α-synuclein (pS129, red) and DAPI (blue). (H) Western blot analysis and quantification of pS129-α-synuclein expression. Data are shown as the mean ± SEM *p < 0.05, **p < 0.01, ***p < 0.001, ****p < 0.0001.

One of the pathophysiological mechanisms associated with PD is oxidative stress, which correlates with an increased generation of ROS and a compromised antioxidant system. The ROS levels within SH-SY5Y cells can be measured through 2,7-dichloro-dihydro-fluorescein diacetate (DCFH-DA) staining analysis. The DCFH-DA fluorescence significantly escalated following cellular exposure to MPP^+^, whereas BAGLPP was able to inhibit ROS production in cells treated with MPP^+^ (Figure 4D,E). In accordance with Figure 4D, ROS-positive cells arose from a baseline of 4.04 ± 0.43% in the control group to 16.08 ± 1.86% within the MPP^+^&BALPP-treated group, but subsequently decreased to 7.39 ± 0.61% when SH-SY5Y cells were pretreated with BAGLPP (Figure 4F). Consequently, BAGLPP demonstrated the capability to lower ROS levels in SH-SY5Y cells elicited by MPP^+^. The excessive accumulation of α-synuclein represents a prominent biomarker for PD pathology, which can be replicated through MPP^+^-induced SH-SY5Y cells. Specifically, phosphorylated α-synuclein at serine 129 (α-syn pS129) is a dominant pathological modification closely linked to aggregation, toxicity, and Lewy body formation in Parkinson’s disease.^36,37^ The immunofluorescence images showed that the cells in the MPP^+^&BALPP group exhibited a higher level of α-syn pS129 and more α-synuclein aggregation signals. However, pretreatment with BAGLPP significantly reduces the levels of α-syn (pS129) induced by MPP^+^ exposure, compared to empty vector groups (Figure 4G). Moreover, the results from Western blot analysis depicted in Figure 4H indicate that the GDNF, delivered via BAGLPP, effectively reduced the level of phosphorylation of α-synuclein.

### 3.5 BAGLPP Prevented Motor Deficits and PD Pathology in MPTP Mice

The in vivo therapeutic potential of BAGLPP was evaluated in an MPTP mouse model. The mice were randomly assigned to four distinct treatment groups: (1) normal control mice receiving saline (Sham), (2) untreated PD mice (MPTP), (3) PD mice administered with BAGLPP (BAGLPP), and (4) PD mice administered with BALPP (BALPP). Saline, BALPP, and BAGLPP were administered intravenously once daily for a seven-day period. Three weeks following the final administration of LPP, PD symptoms were induced via five intraperitoneal administrations of MPTP (Figure 5A). Following a seven-day period, behavioral assessments were conducted to evaluate the protective effects of BAGLPP against motor dysfunction resulting from MPTP administration. In the open-field test, PD mice treated with BAGLPP exhibited motion trajectories comparable to those in the Sham group, with motor behaviors significantly ameliorated relative to PD mice either untreated or treated with BALPP (Figure 5B). Quantitative analyses of total distance and immobile time further substantiate that BAGLPP significantly mitigated the impairments induced by MPTP in mice (Figure 5C,D). In comparison to normal mice, MPTP-treated mice demonstrated a significantly reduced duration on the rotating rod, which can be attributed to PD-related cerebral damage. Notwithstanding, PD mice treated with BAGLPP demonstrated an average duration of 222.6 ± 22.3 s on the rod, surpassing untreated mice by 82.4 ± 27.8 s (Figure 5E). Conversely, this improvement was not observed in the BALPP treatment group, where rotarod performance remained unchanged compared with untreated PD mice. These behavioral findings indicate that BAGLPP constitutes an efficacious intervention for the prevention of PD motor symptoms.

**Figure 5:**
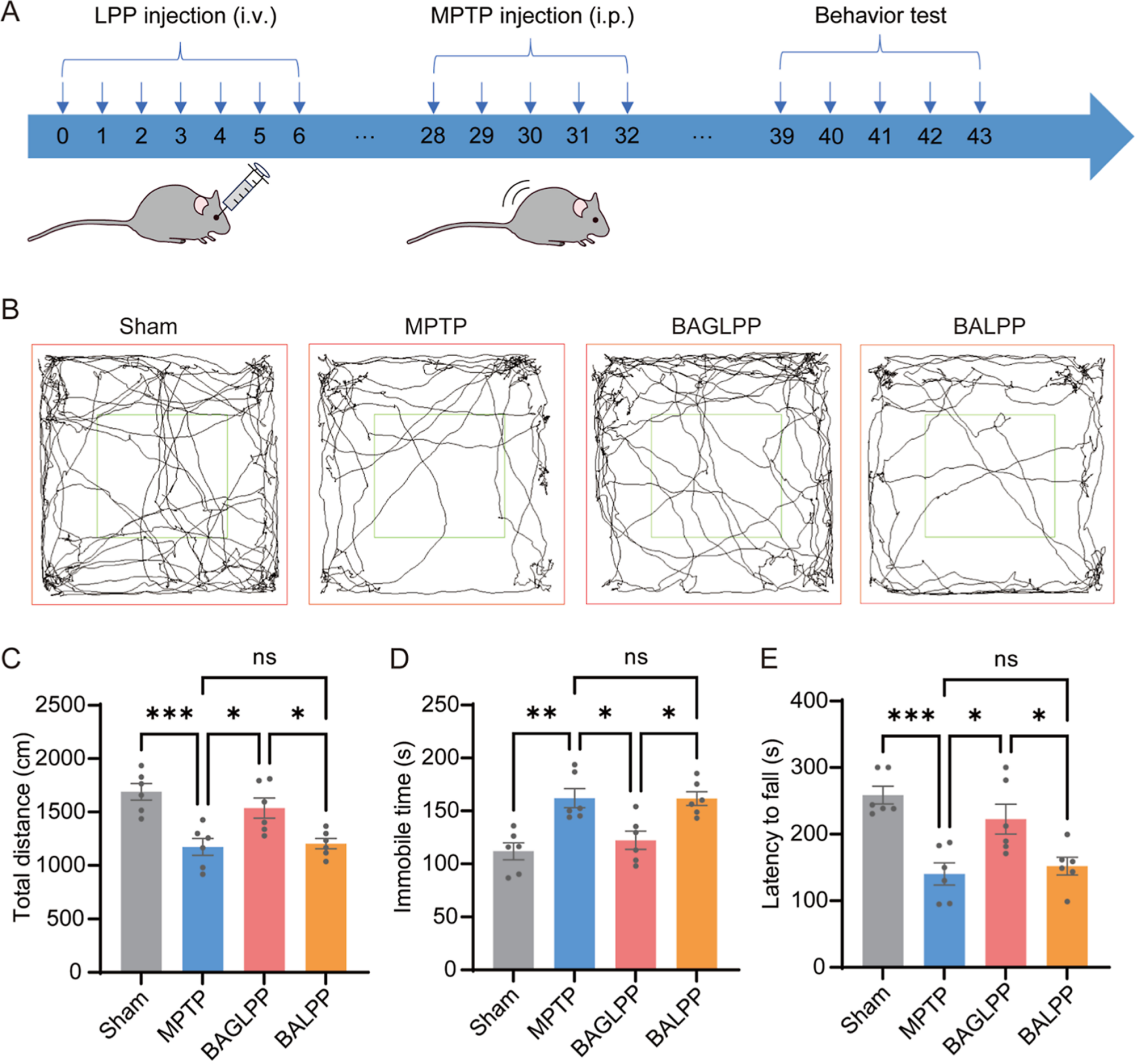
BAGLPP improves motor performance in MPTP-induced PD mice. (A) Schematic timeline of LPP intravenous (i.v.) administration and MPTP intraperitoneal (i.p.) injection for PD model establishment. (B) Representative movement tracks of mice from each group in the open-field test. (C) Total distance traveled in the open field (*n* = 6 per group). (D) Immobility time in the open field (*n* = 6 per group). (E) Latency to fall in the accelerating rotarod test (*n* = 6 per group). Data are presented as mean ± SEM *p < 0.05, **p < 0.01, ***p < 0.001; ns, not significant.

The pathognomonic hallmark of PD is the neurodegeneration of dopaminergic neurons within the SNpc. Tyrosine hydroxylase (TH) serves as the rate-limiting enzyme in dopamine (DA) biosynthesis, and its dysfunction may contribute to the loss of DA neurons in PD. Accordingly, we investigated the influence of BAGLPP on TH levels following MPTP treatment.

After 45 days of in vivo experiment tests, mice from the four treatment groups were sacrificed, and then their blood and major organs were extracted for further analysis. Immunofluorescence analysis revealed that the number of viable dopaminergic neurons, identified as tyrosine hydroxylase-positive (TH^+^) in the substantia nigra, was significantly increased when treated with BAGLPP in comparison to that in the MPTP group (Figure 6A,B). Additionally, we evaluated the DA concentrations in the striatum across different treatment groups. Subsequent to the loss of approximately 50% of dopaminergic neurons following MPTP injection (Figure 6B), only 25% of the DA level in the striatum persisted in PD mice. Notably, treatment with BAGLPP enabled the recovery of DA levels in the striatum of PD mice from 60% (Figure 6C). In contrast, no significant protective effect was observed in the BALPP treatment group, highlighting the substantial neuroprotective effect of the BAGLPP in PD intervention. Neuroinflammation is vital in the progression of PD. Analysis of cytokines from brain tissues indicated that BAGLPP-treated PD mice exhibited reduced levels of proinflammatory cytokines, such as tumor necrosis factor-α (TNF-α) and interleukin-6 (IL-6) (Figure 6D,E). Consistent with cellular findings, the α-syn (pS129) levels in the brain tissue were notably lower in the BAGLPP treatment group than in the MPTP and BALPP treatment groups (Figure 6F,G). These preliminary findings underscore the potential of the BAGLPP in providing neuroprotection and preventing PD.

**Figure 6:**
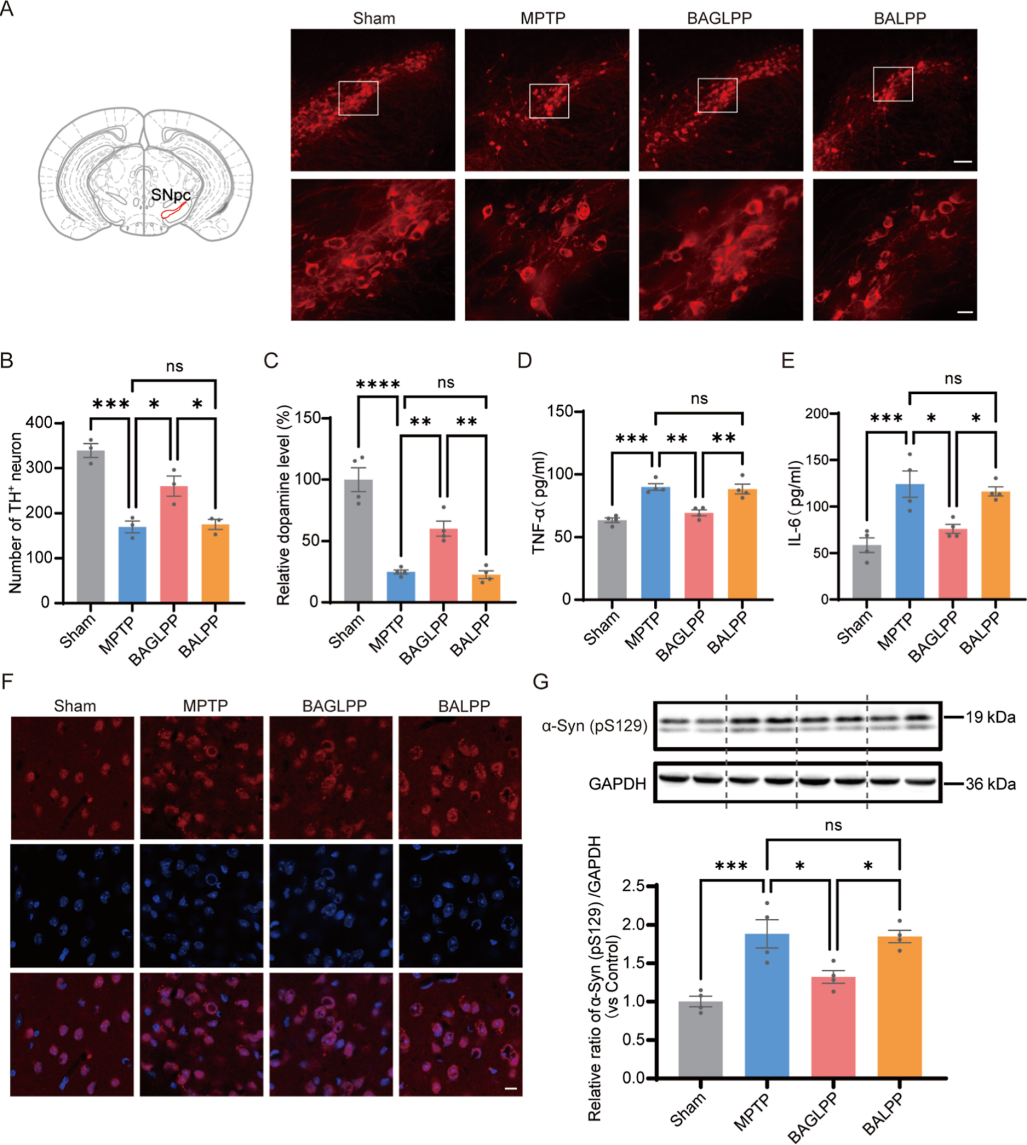
BAGLPP protects against neuropathology in MPTP-treated mice. (A) Representative immunofluorescence images of TH-positive dopaminergic neurons in the substantia nigra pars compacta (SNpc) for each group. The schematic (left) indicates the SNpc location (red line; adapted from Allen Mouse Brain Atlas). Upper panels: maximum-intensity projections (scale bar: 100 μm). Lower panels: magnified views (scale bar: 20 μm). (B) Quantification of surviving TH^+^ neurons in the SNpc (*n* = 3). (C) Striatal dopamine levels across groups (*n* = 4). (D,E) Striatal levels of pro-inflammatory cytokines TNF-α and IL-6 (*n* = 4). (F) Representative immunofluorescence of phosphorylated α-synuclein (pS129, red) and DAPI (blue) in the striatum. (G) Western blot and quantification of pS129 α-synuclein expression in the striatum (*n* = 4). Data are shown as mean ± SEM *p < 0.05, **p < 0.01, ***p < 0.001, ****p < 0.0001; ns, not significant.

### 3.6 Sustained GDNF Expression and Mechanistic Insights of BAGLPP

To definitively establish the capacity of the BAGLPP platform for sustained functional expression under neurodegenerative stress, we performed a dedicated prophylactic study. Mice received a series of intravenous injections of either BAGLPP or BALPP. After a 3 week period to allow for stable transgene expression, all animals were subjected to the subacute MPTP regimen. Striatal tissues were harvested at 1, 4, and 8 weeks post-MPTP modeling for quantification of GDNF levels (Figure 7A).

**Figure 7:**
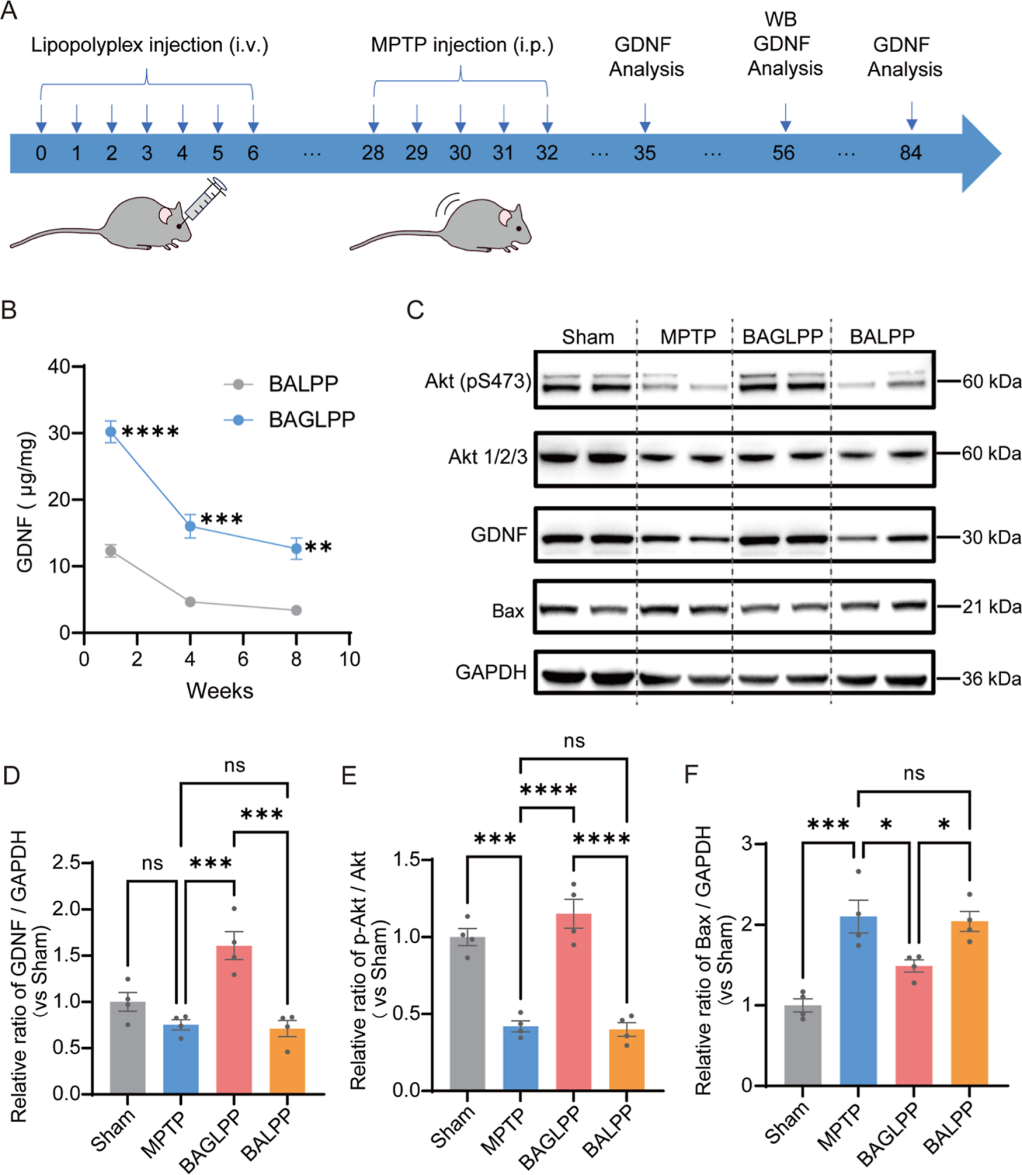
Sustained GDNF expression and regulation of neuroprotective pathways by BAGLPP in MPTP-treated mice. (A) Experimental timeline of intravenous (i.v.) LPP administration and intraperitoneal (i.p.) MPTP injections and analysis time points. (B) GDNF protein levels in the striatum at the indicated times post-treatment (*n* = 4). (C) Representative Western blot of *p*-Akt (S473), total Akt, GDNF, and Bax in striatal lysates. (D–F) Quantification of the GDNF (D), *p*-Akt/Akt ratio (E), and Bax (F) expression (*n* = 4). Data are mean ± SEM *p < 0.05, **p < 0.01, ***p < 0.001, ****p < 0.0001; ns, not significant.

In BALPP-pretreated control mice, GDNF levels exhibited a gradual decline following MPTP lesioning, consistent with the exhaustion of endogenous compensatory mechanisms under prolonged pathological stress. In striking contrast, BAGLPP pretreatment established and maintained significantly elevated levels of the GDNF in the brain after the MPTP challenge. Although a long-term decline was observed, the exogenous GDNF expression remained at a relatively high level throughout the neurotoxic insult compared to that in the BALPP control group. Notably, at 8 weeks post-MPTP, GDNF levels in the BAGLPP group were 3.7-fold higher than those in the BALPP group (Figure 7B). These results demonstrate that BAGLPP enables long-term GDNF expression, providing continuous trophic support to help neurons resist degenerative pressure.

To directly elucidate the molecular mechanism underlying this neuroprotection, we analyzed key protein markers in the striatum. Pretreatment with BAGLPP effectively sustained high GDNF protein levels after MPTP lesion (Figure 7B–D). Concomitantly, this elevated GDNF expression enhanced the phosphorylation of Akt at Ser473 (*p*-Akt), a central node in pro-survival signaling (Figure 7C,E). The increased *p*-Akt/Akt ratio indicates potent activation of the PI3K/Akt pathway.^38^ In parallel, the pro-apoptotic signal was robustly suppressed, as evidenced by a significant reduction in the level of the pro-apoptotic protein Bax in the BAGLPP-treated group compared to the MPTP model group (Figure 7C,F).

The in vivo safety of LPP was further assessed by routine blood tests and biochemistry. We found that the blood routine and most serum biochemistry parameters showed similarities between the control and treatment groups (Supporting Information Figure 3). However, the globulin level and albumin/globulin ratio of BALPP groups decreased compared to those of the control, which might be due to the combined toxicity of MPTP and LPP. Interestingly, compared to BALPP, BAGLPP demonstrated a protective effect on globulin levels and albumin/globulin ratio (Supporting Information Figure S3G,H).

## 4 Discussion

The rising global incidence of Parkinson’s disease (PD) highlights the urgent need for disease-modifying therapies. Although the glial cell line-derived neurotrophic factor (GDNF) has shown therapeutic promise, its clinical translation has been limited by poor blood–brain barrier (BBB) penetration and challenges in timing intervention. Here, we developed a brain-targeted, nonviral gene delivery platform—BAGLPP—that enables sustained, site-specific GDNF expression after intravenous administration. Our results demonstrate that BAGLPP provides comprehensive neuroprotection, attenuating dopaminergic neuron loss, neuroinflammation, and pathological α-synuclein accumulation in a preclinical PD model.

The efficacy of BAGLPP stems from its rationally designed lipopolyplex (LPP) architecture, which synergistically enhances transfection efficiency and brain-specific targeting. First, employing an AAV-derived plasmid backbone promoted persistent transgene expression, achieving >30% transfection in SH-SY5Y cells and sustaining GDNF production for up to 8 weeks postlesion. Second, surface conjugation of the RVG29 peptide enabled active brain targeting via binding to nAChR on cerebrovascular endothelial cells, facilitating receptor-mediated transcytosis across the BBB.^25,39–43^ Our results demonstrate that inhibiting the clathrin-mediated endocytosis in SH-SY5Y cells significantly reduces the transfection efficiency of BAELPP, which correlated with the high expression level of nAChR in SH-SY5Y cells.^26,44,45^ Biodistribution studies confirmed significantly higher brain transduction compared with nontargeted controls, underscoring the importance of targeted delivery for maximizing therapeutic efficacy while minimizing off-target effects.

Our study focuses on neuroprotection by administering the GDNF prior to nigrostriatal lesioning. While neurorestorative approaches after significant neuronal loss remain challenging,^46,47^ BAGLPP delivered before lesion induction robustly preserved motor function in MPTP-treated mice. Mechanistically, BAGLPP-mediated GDNF expression activated the pro-survival PI3K/Akt pathway and suppressed apoptosis (reduced Bax), thereby protecting dopaminergic neurons. Notably, the phosphorylation status of tyrosine hydroxylase (pTH) provides a sensitive readout of acute dopaminergic neuron activity,^48^ future studies investigating dynamic changes in pTH could further elucidate the acute adaptive responses of rescued neurons to GDNF signaling.

Beyond direct neuronal protection, our strategy effectively counteracts the core pathologies of PD. BAGLPP significantly reduced phosphorylated α-synuclein (pS129) levels.^36,37^ This suggests that GDNF signaling may enhance cellular mechanisms for clearing misfolded proteins.^49,50^ Furthermore, some researchers have found that increasing the level of the GDNF can enhance the activity of dopamine transporters.^51,52^ Concurrently, the dampening of neuroinflammation, indicated by reduced levels of TNF-α and IL-6, helps to break the vicious cycle where inflammation begets further neuronal damage.^53^ Together, these effects preserved nigrostriatal integrity, restored striatal dopamine, and improved motor performance.

Compared with existing delivery systems, BAGLPP offers distinct advantages. AAVs are the clinical frontrunners for CNS gene therapy, offering potential long-term expression.^54,55^ However, they are hampered by pre-existing immunity, limited cargo capacity, high manufacturing costs, and the challenges associated with redosing.^56^ Indeed, the widespread transfection level of LPP in the brain via intravenous injection was much lower than that of AAV-PHP.B or AAV-PHP.eB.^57,58^ Recent research showed GDNF overexpression in the midbrain may lead to side effects,^50^ indicating that a moderate expression of the GDNF may have better therapeutic effects. Lipid nanoparticles (LNPs) are currently the most widely used nucleic acid drug delivery carrier in clinical applications;^59–61^ while revolutionary for systemic mRNA delivery, they exhibit strong tropism for the liver and require sophisticated targeting ligands to achieve significant brain delivery.^62–64^ Exosomes, or natural extracellular vesicles, show innate biocompatibility and BBB-crossing potential but face challenges in scalable production, consistent drug loading, and functionalization.^65,66^ BAGLPP thus occupies a unique niche, combining the safety and cargo flexibility of nonviral vectors with the targeting precision needed for CNS applications.

The translational potential of BAGLPP is supported by its efficacy following intravenous administration and a favorable preliminary safety profile. Its preventive effects in the MPTP model suggest promise for early-stage or presymptomatic PD patients. Future work should include long-term toxicology studies in higher species, scalable GMP-compliant manufacturing, and validation in chronic or genetic PD models. Optimization of the dosing regimens will also be essential to maintain therapeutic GDNF levels over the prolonged course of PD.

In summary, we have developed a brain-targeted lipopolyplex that enables sustained GDNF gene delivery, providing multifaceted neuroprotection in a preclinical PD model. By achieving durable gene expression, activating pro-survival pathways, and mitigating key PD pathologies, BAGLPP represents a promising nonviral platform not only for PD but potentially also for other neurodegenerative disorders.

## Supplementary Material



## Data Availability

All data associated with this study are present in the paper.

## Notes

Gel retardation assay of polyplex and lipopolyplex formulations; serum nuclease protection assay; serum biochemistry analysis (ALT, AST, creatinine, urea, total protein, albumin, globulin, albumin/globulin ratio); physicochemical characteristics of polyplexes; liposomes, and lipopolyplexes (size, PDI, zeta potential); and antibody information (applications, dilutions, sources) (PDF)

## References

[1] Dorsey E. R., Sherer T., Okun M. S., Bloem B. R. (2018). The Emerging Evidence
of the Parkinson Pandemic. J. Park. Dis..

[2] Poewe W., Seppi K., Tanner C. M., Halliday G. M., Brundin P., Volkmann J., Schrag A.-E., Lang A. E. (2017). Parkinson Disease. Nat. Rev.
Dis. Primer.

[3] Connolly B. S., Lang A. E. (2014). Pharmacological
Treatment of Parkinson Disease: A Review. JAMA.

[4] Armstrong M. J., Okun M. S. (2020). Diagnosis and Treatment
of Parkinson Disease: A Review. JAMA.

[5] Church F. C. (2021). Treatment
Options for Motor and Non-Motor Symptoms of Parkinson’s Disease. Biomolecules.

[6] Janssen
Daalen J. M., Schootemeijer S., Richard E., Darweesh S. K. L., Bloem B. R. (2022). Lifestyle Interventions for the Prevention of Parkinson
Disease: A Recipe for Action. Neurology.

[7] Petzinger G. M., Fisher B. E., McEwen S., Beeler J. A., Walsh J. P., Jakowec M. W. (2013). Exercise-Enhanced
Neuroplasticity Targeting Motor and
Cognitive Circuitry in Parkinson’s Disease. Lancet Neurol..

[8] Wang X., Fu S., Yoo K., Wang X., Gan L., Zou T., Gao Q., Han H., Yang Z., Hu X., Chen H., Liu D., Li R. (2024). Individualized Structural
Perturbations on Normative
Brain Connectome Restrict Deep Brain Stimulation Outcomes in Parkinson’s
Disease. Mov. Disord. Off. J. Mov. Disord. Soc..

[9] Cernera S., Eisinger R. S., Wong J. K., Ho K. W. D., Lopes J. L., To K., Carbunaru S., Ramirez-Zamora A., Almeida L., Foote K. D., Okun M. S., Gunduz A. (2020). Long-Term Parkinson’s Disease
Quality of Life after Staged DBS: STN vs GPi and First vs Second Lead. NPJ. Park. Dis..

[10] Sharma V. D., Patel M., Miocinovic S. (2020). Surgical Treatment
of Parkinson’s
Disease: Devices and Lesion Approaches. Neurother.
J. Am. Soc. Exp. Neurother..

[11] Lin L. F., Doherty D. H., Lile J. D., Bektesh S., Collins F. (1993). GDNF: A Glial
Cell Line-Derived Neurotrophic Factor for Midbrain Dopaminergic Neurons. Science.

[12] Beck K. D., Valverde J., Alexi T., Poulsen K., Moffat B., Vandlen R. A., Rosenthal A., Hefti F. (1995). Mesencephalic Dopaminergic
Neurons Protected by GDNF from Axotomy-Induced Degeneration in the
Adult Brain. Nature.

[13] Gash D. M., Zhang Z., Ovadia A., Cass W. A., Yi A., Simmerman L., Russell D., Martin D., Lapchak P. A., Collins F., Hoffer B. J., Gerhard G. A. (1996). Functional Recovery
in Parkinsonian Monkeys Treated with GDNF. Nature.

[14] Eslamboli A., Georgievska B., Ridley R. M., Baker H. F., Muzyczka N., Burger C., Mandel R. J., Annett L., Kirik D. (2005). Continuous
Low-Level Glial Cell Line-Derived Neurotrophic Factor Delivery Using
Recombinant Adeno-Associated Viral Vectors Provides Neuroprotection
and Induces Behavioral Recovery in a Primate Model of Parkinson’s
Disease. J. Neurosci. Off. J. Soc. Neurosci..

[15] Björklund A., Kirik D., Rosenblad C., Georgievska B., Lundberg C., Mandel R. J. (2000). Towards a Neuroprotective
Gene Therapy
for Parkinson’s Disease: Use of Adenovirus, AAV and Lentivirus
Vectors for Gene Transfer of GDNF to the Nigrostriatal System in the
Rat Parkinson Model. Brain Res..

[16] Hamilton B. A., Wright J. F. (2021). Challenges Posed
by Immune Responses to AAV Vectors:
Addressing Root Causes. Front. Immunol..

[17] Barker R. A., Björklund A., Gash D. M., Whone A., Van Laar A., Kordower J. H., Bankiewicz K., Kieburtz K., Saarma M., Booms S., Huttunen H. J., Kells A. P., Fiandaca M. S., Stoessl A. J., Eidelberg D., Federoff H., Voutilainen M. H., Dexter D. T., Eberling J., Brundin P., Isaacs L., Mursaleen L., Bresolin E., Carroll C., Coles A., Fiske B., Matthews H., Lungu C., Wyse R. K., Stott S., Lang A. E. (2020). GDNF and Parkinson’s Disease:
Where Next? A Summary from a Recent Workshop. J. Park. Dis..

[18] d’Anglemont de Tassigny, X. ; Pascual, A. ; López-Barneo, J. GDNF-Based Therapies, GDNF-Producing Interneurons, and Trophic Support of the Dopaminergic Nigrostriatal Pathway. Implications for Parkinson’s Disease. Front. Neuroanat. 2015, 9. 10.3389/fnana.2015.00010 PMC4327623 25762899

[19] Yin H., Kanasty R. L., Eltoukhy A. A., Vegas A. J., Dorkin J. R., Anderson D. G. (2014). Non-Viral
Vectors for Gene-Based Therapy. Nat. Rev. Genet..

[20] Chen, W. ; Li, H. ; Liu, Z. ; Yuan, W. Lipopolyplex for Therapeutic Gene Delivery and Its Application for the Treatment of Parkinson’s Disease. Front. Aging Neurosci. 2016, 8. 10.3389/fnagi.2016.00068 PMC4820442 27092073

[21] Rezaee M., Oskuee R. K., Nassirli H., Malaekeh-Nikouei B. (2016). Progress in
the Development of Lipopolyplexes as Efficient Non-Viral Gene Delivery
Systems. J. Control. Release Off. J. Control.
Release Soc..

[22] Pardridge W. M. (2020). Brain Delivery
of Nanomedicines: Trojan Horse Liposomes for Plasmid DNA Gene Therapy
of the Brain. Front. Med. Technol..

[23] Wang C., Xue Y., Markovic T., Li H., Wang S., Zhong Y., Du S., Zhang Y., Hou X., Yu Y., Liu Z., Tian M., Kang D. D., Wang L., Guo K., Cao D., Yan J., Deng B., McComb D. W., Parsons R. E., Minier-Toribio A. M., Holt L. M., Pan J., Hashemi A., Kopell B. H., Charney A. W., Nestler E. J., Peng P. C., Dong Y. (2025). Blood-Brain-Barrier-Crossing
Lipid Nanoparticles for mRNA Delivery
to the Central Nervous System. Nat. Mater..

[24] Kumar P., Wu H., McBride J. L., Jung K.-E., Hee Kim M., Davidson B. L., Kyung Lee S., Shankar P., Manjunath N. (2007). Transvascular
Delivery of Small Interfering RNA to the Central Nervous System. Nature.

[25] Gong C., Li X., Xu L., Zhang Y.-H. (2012). Target Delivery of a Gene into the
Brain Using the RVG29-Oligoarginine Peptide. Biomaterials.

[26] Gotti C., Clementi F. (2004). Neuronal Nicotinic Receptors: From Structure to Pathology. Prog. Neurobiol..

[27] Qu M., Lin Q., He S., Wang L., Fu Y., Zhang Z., Zhang L. (2018). A Brain Targeting
Functionalized Liposomes of the Dopamine Derivative
N −3,4-Bis­(Pivaloyloxy)-Dopamine for Treatment of Parkinson’s
Disease. J. Controlled Release.

[28] Liu Y., Huang R., Han L., Ke W., Shao K., Ye L., Lou J., Jiang C. (2009). Brain-Targeting
Gene Delivery and
Cellular Internalization Mechanisms for Modified Rabies Virus Glycoprotein
RVG29 Nanoparticles. Biomaterials.

[29] Earley L. F., Conatser L. M., Lue V. M., Dobbins A. L., Li C., Hirsch M. L., Samulski R. J. (2020). Adeno-Associated
Virus Serotype-Specific
Inverted Terminal Repeat Sequence Role in Vector Transgene Expression. Hum. Gene Ther..

[30] Zhang H. (2017). Thin-Film
Hydration Followed by Extrusion Method for Liposome Preparation. Methods Mol. Biol. Clifton NJ..

[31] Schäfer J., Höbel S., Bakowsky U., Aigner A. (2010). Liposome-Polyethylenimine
Complexes for Enhanced DNA and siRNA Delivery. Biomaterials.

[32] Gong J., Wang H.-X., Leong K. W. (2019). Determination of Cellular Uptake
and Endocytic Pathways. Bio-Protoc..

[33] Jackson-Lewis V., Przedborski S. (2007). Protocol for the MPTP Mouse Model
of Parkinson’s
Disease. Nat. Protoc..

[34] Sang Y., Xie K., Mu Y., Lei Y., Zhang B., Xiong S., Chen Y., Qi N. (2015). Salt Ions
and Related Parameters
Affect PEI-DNA Particle Size and Transfection Efficiency in Chinese
Hamster Ovary Cells. Cytotechnology.

[35] Gong P., Deng F., Zhang W., Ji J., Liu J., Sun Y., Hu J. (2017). Tectorigenin Attenuates
the MPP+-Induced SH-SY5Y Cell
Damage, Indicating a Potential Beneficial Role in Parkinson’s
Disease by Oxidative Stress Inhibition. Exp.
Ther. Med..

[36] Choi S. G., Tittle T., Garcia-Prada D., Kordower J. H., Melki R., Killinger B. A. (2024). Alpha-Synuclein Aggregates Are Phosphatase Resistant. Acta Neuropathol. Commun..

[37] Oueslati A. (2016). Implication
of Alpha-Synuclein Phosphorylation at S129 in Synucleinopathies: What
Have We Learned in the Last Decade?. J. Park.
Dis..

[38] Khan M. N., Choudhary D., Mehan S., Khan Z., Gupta G. D., Narula A. S. (2025). Molecular
Mechanisms of GDNF/GFRA1/RET and PI3K/AKT/ERK
Signaling Interplay in Neuroprotection: Therapeutic Strategies for
Treating Neurological Disorders. Neuropeptides.

[39] Gan L., Li Z., Lv Q., Huang W. (2019). Rabies Virus Glycoprotein
(RVG29)-Linked
microRNA-124-Loaded Polymeric Nanoparticles Inhibit Neuroinflammation
in a Parkinson’s Disease Model. Int.
J. Pharm..

[40] Jiang P., Xiao Y., Hu X., Wang C., Gao H., Huang H., Lv J., Qi Z., Wang Z. (2024). RVG29 Peptide-Modified
Exosomes Loaded with Mir-133b Mediate the RhoA-ROCK Pathway to Improve
Motor and Neurological Symptoms in Parkinson’s Disease. ACS Biomater. Sci. Eng..

[41] Dang Y., Shen F., Wang S., Zhang Y., Lu X., Qin D., Feng D., Song Y., Cheng Z., Ma R., Wang F. (2025). Dual-Modified
Mannose/RVG29 Peptide-Functionalized Lipid Nanoparticles
Loaded With circHIPK2 siRNA Ameliorate Hypoxic-Ischemic Brain Damage
in Neonatal Mice by Suppressing Astrocyte Activation. J. Integr. Neurosci..

[42] Wu J.-W., Zhou Y.-T., Wang B.-X., Shen L.-P., Zhang X.-Q., Du Z.-Y., Wang P., Lu X.-J., Miao Z.-L., Zhao X.-D. (2025). Apigenin Regulates
CCR5/JAK1/STAT1/MMPs Signaling to
Alleviate Secondary Brain Injury after Intracerebral Hemorrhage and
Its Enhanced Delivery via Targeted Nanoparticles. J. Nanobiotechnology.

[43] You L., Wang J., Liu T., Zhang Y., Han X., Wang T., Guo S., Dong T., Xu J., Anderson G. J., Liu Q., Chang Y.-Z., Lou X., Nie G. (2018). Targeted Brain Delivery
of Rabies Virus Glycoprotein 29-Modified
Deferoxamine-Loaded Nanoparticles Reverses Functional Deficits in
Parkinsonian Mice. ACS Nano.

[44] Ridley D. L., Rogers A., Wonnacott S. (2001). Differential
Effects of Chronic Drug
Treatment on Alpha3* and Alpha7 Nicotinic Receptor Binding Sites,
in Hippocampal Neurones and SH-SY5Y Cells. Br.
J. Pharmacol..

[45] Dunckley T., Lukas R. J. (2006). Nicotinic Modulation of Gene Expression in SH-SY5Y
Neuroblastoma Cells. Brain Res..

[46] Tenenbaum, L. ; Humbert-Claude, M. Glial Cell Line-Derived Neurotrophic Factor Gene Delivery in Parkinson’s Disease: A Delicate Balance between Neuroprotection, Trophic Effects, and Unwanted Compensatory Mechanisms. Front. Neuroanat. 2017, 11. 10.3389/fnana.2017.00029 PMC5385337 28442998

[47] Francardo V., Schmitz Y., Sulzer D., Cenci M. A. (2017). Neuroprotection
and Neurorestoration as Experimental Therapeutics for Parkinson’s
Disease. Exp. Neurol..

[48] Johnson M. E., Salvatore M. F., Maiolo S. A., Bobrovskaya L. (2018). Tyrosine Hydroxylase
as a Sentinel for Central and Peripheral Tissue Responses in Parkinson’s
Progression: Evidence from Clinical Studies and Neurotoxin Models. Prog. Neurobiol..

[49] Chen J., Ling Z., Lv M., Chen S., Xu W., Shao L., Ma Z., Wang C., Song Y., Tang C. (2025). GDNF Attenuates A-Synuclein
Aggregation-Induced Damage to VTA-NAc
Dopaminergic Transmission and Alleviates Depression-like Behaviors
in Mice. Sci. Rep..

[50] Er S., Parkkinen I., Trepczyk K., Seelbach A., Pasculli M. S., De Lorenzo F., Luk K., Jankowska E., Chmielarz P., Domanskyi A., Airavaara M. (2025). GDNF Reduces
Fibril-Induced Early-Stage Alpha-Synuclein Pathology after Delivery
of 20S Proteasome Inhibitor Lactacystin. Eur.
J. Pharm. Sci. Off. J. Eur. Fed. Pharm. Sci..

[51] Littrell O. M., Pomerleau F., Huettl P., Surgener S., McGinty J. F., Middaugh L. D., Granholm A.-C., Gerhardt G. A., Boger H. A. (2012). Enhanced
Dopamine Transporter Activity in Middle-Aged Gdnf Heterozygous Mice. Neurobiol. Aging.

[52] Chengcheng M., Panpan A., Yalong Y., Mingyu S., Wei X., Jing C., Chuanxi T. (2024). GDNF Improves the Cognitive Ability
of PD Mice by Promoting Glycosylation and Membrane Distribution of
DAT. Sci. Rep..

[53] Araújo B., Caridade-Silva R., Soares-Guedes C., Martins-Macedo J., Gomes E. D., Monteiro S., Teixeira F. G. (2022). Neuroinflammation
and Parkinson’s Disease—From Neurodegeneration to Therapeutic
Opportunities. Cells.

[54] Kang L., Jin S., Wang J., Lv Z., Xin C., Tan C., Zhao M., Wang L., Liu J. (2023). AAV Vectors Applied
to the Treatment of CNS Disorders: Clinical Status and Challenges. J. Control. Release Off. J. Control. Release Soc..

[55] Yin L., He H., Zhang H., Shang Y., Fu C., Wu S., Jin T. (2025). Revolution
of AAV in Drug Discovery: From Delivery System to Clinical
Application. J. Med. Virol..

[56] Huang L., Wan J., Wu Y., Tian Y., Yao Y., Yao S., Ji X., Wang S., Su Z., Xu H. (2021). Challenges in Adeno-Associated
Virus-Based Treatment of Central Nervous System Diseases through Systemic
Injection. Life Sci..

[57] Deverman B. E., Pravdo P. L., Simpson B. P., Kumar S. R., Chan K. Y., Banerjee A., Wu W.-L., Yang B., Huber N., Pasca S. P., Gradinaru V. (2016). Cre-Dependent Selection Yields AAV
Variants for Widespread Gene Transfer to the Adult Brain. Nat. Biotechnol..

[58] Carneiro A. D., Schaffer D. V. (2024). Engineering Novel Adeno-Associated Viruses (AAVs) for
Improved Delivery in the Nervous System. Curr.
Opin. Chem. Biol..

[59] Wang B., Shen B., Xiang W., Shen H. (2024). Advances in the Study
of LNPs for mRNA Delivery and Clinical Applications. Virus Genes.

[60] Hou X., Zaks T., Langer R., Dong Y. (2021). Lipid Nanoparticles
for mRNA Delivery. Nat. Rev. Mater..

[61] Ma R., Li Y., Su Y., Chen P., Xie S., Tan W., Liu X. (2025). Lipid Nanoparticles:
A Delicate Nucleic Acid Delivery System to Be
Further Explored. Nano Today.

[62] Cheng Q., Wei T., Farbiak L., Johnson L. T., Dilliard S. A., Siegwart D. J. (2020). Selective
Organ Targeting (SORT) Nanoparticles for Tissue-Specific mRNA Delivery
and CRISPR-Cas Gene Editing. Nat. Nanotechnol..

[63] Han E. L., Tang S., Kim D., Murray A. M., Swingle K. L., Hamilton A. G., Mrksich K., Padilla M. S., Palanki R., Li J. J., Mitchell M. J. (2025). Peptide-Functionalized
Lipid Nanoparticles
for Targeted Systemic mRNA Delivery to the Brain. Nano Lett..

[64] Khare P., Edgecomb S. X., Hamadani C. M., Tanner E. E. L., S
Manickam D. (2023). Lipid Nanoparticle-Mediated Drug Delivery to the Brain. Adv. Drug Delivery Rev..

[65] Mehdizadeh S., Mamaghani M., Hassanikia S., Pilehvar Y., Ertas Y. N. (2025). Exosome-Powered
Neuropharmaceutics: Unlocking the Blood-Brain Barrier for next-Gen
Therapies. J. Nanobiotechnology.

[66] Yang C., Xue Y., Duan Y., Mao C., Wan M. (2024). Extracellular Vesicles
and Their Engineering Strategies, Delivery Systems, and Biomedical
Applications. J. Control. Release Off. J. Control.
Release Soc..

