# Supplementary material for: Advancing Parkinson’s Prevention: Brain-Specific Lipopolyplex Delivery of the GDNF as a Neuroprotective Gene Therapy
Source: ACS Mater Au. 2026 Mar 24;6(3):642–56. doi: 10.1021/acsmaterialsau.6c00024 (PMC13177410; doi:10.1021/acsmaterialsau.6c00024)
Supplement: Supplementary file 1 [file mg6c00024_si_001.pdf]

Supporting Information

Advancing Parkinson’s Prevention: Brain-Specific Lipopolyplex Delivery of the GDNF as a Neuroprotective Gene Therapy

Yujing Zhang, Yujing Huang, Zhonghua Lu, Zhen Yuan.

List of Content

Supplementary Figures 1-3

Supplementary Tables 1-2

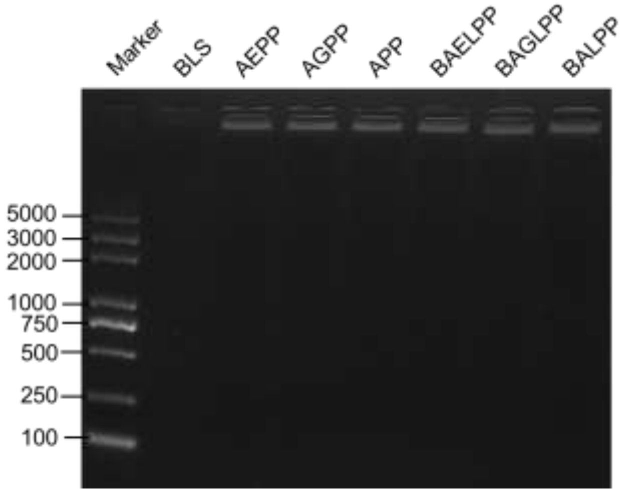

Supplementary Figure 1. Gel retardation assay of various polyplex and lipopolyplex formulations.

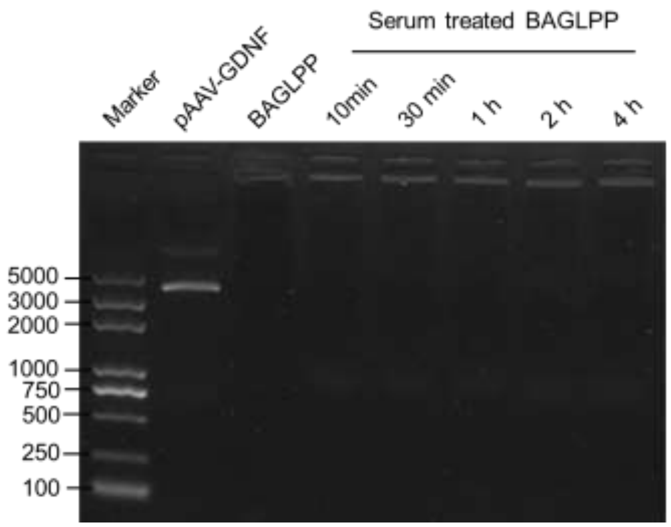

Supplementary Figure 2. Serum nuclease protection assay.

BAGLPP effectively protected plasmid DNA from degradation by serum nucleases. DNA loading: 100 ng per lane.

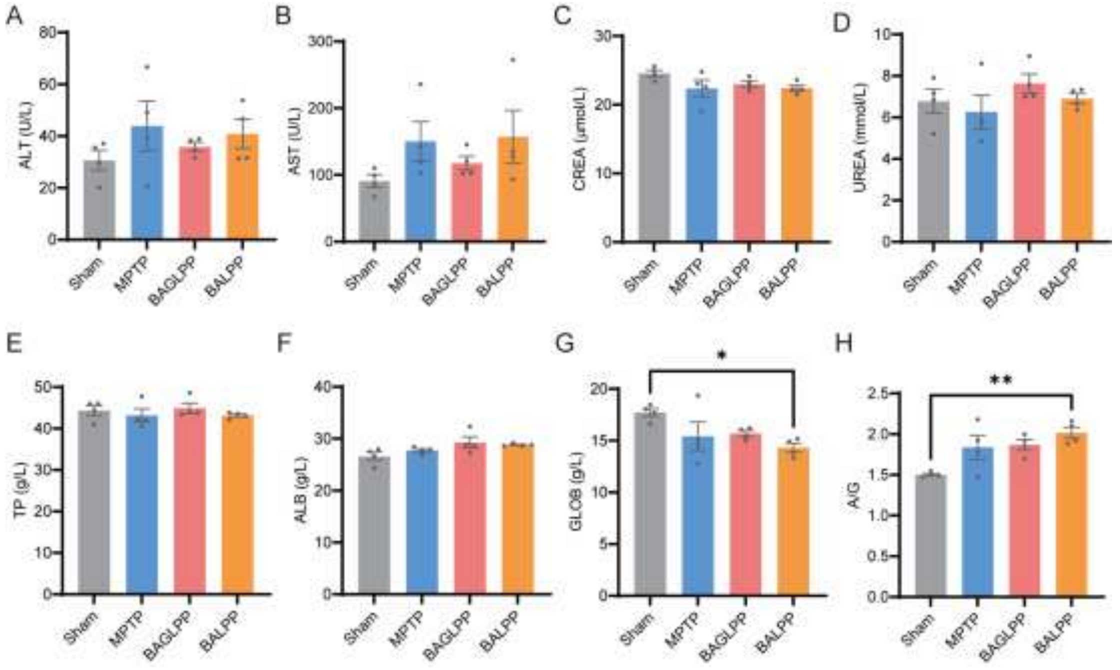

Supplementary Figure 3. Serum biochemistry analysis.

Levels of (A) alanine aminotransferase (ALT), (B) aspartate aminotransferase (AST), (C) creatinine, (D) urea, (E) total protein, (F) albumin, (G) globulin, and (H) albumin/globulin ratio across treatment groups (n = 4).

Data are shown as mean ± SEM. Statistical significance was assessed versus the sham group. *$p < 0.05$, **$p < 0.01$; absence of bars indicates no significant difference.

Supplementary Table 1 Physicochemical characteristics of polyplexes, liposomes and lipopolyplexes

Name	Description	Size ± SEM (nm)	PDI ± SEM	Zeta potential ± SEM (mV)
AEPP	pAAV-EYFP polyplex	>1000	1	16.89 ± 0.58
AGPP	pAAV-GDNF polyplex	>1000	1	15.90 ± 0.43
APP	pAAV control polyplex	>1000	1	16.87 ± 0.62
LS	Liposome (DPPC/DSPE-PEG2000/ Cholesterol)	165.7 ± 0.4	0.166 ± 0.008	-5.94 ± 0.31
AELPP	pAAV-EYFP lipopolyplex (AEPP+LS)	165.4 ± 2.2	0.192 ± 0.016	7.53 ± 0.25
BLS	Brain-targeting liposome (DPPC/DSPE-PEG2000-RVG29/Cholesterol)	208.1 ± 3.6	0.153 ± 0.007	-3.62 ± 0.16
BAELPP	Brain-targeting pAAV-EYFP lipopolyplex (AEPP+BLS)	186.9 ± 2.3	0.221 ± 0.011	9.28 ± 0.35
BAGLPP	Brain-targeting pAAV-GDNF lipopolyplex (AGPP+BLS)	185.5 ± 1.2	0.168 ± 0.011	8.23 ± 0.25
BALPP	Brain-targeting pAAV control lipopolyplex (APP+BLS)	179.5 ± 3.4	0.174 ± 0.006	8.19 ± 0.27

PDI: Polydispersity Index

Supplementary Table 2. Information of antibody used in this study

Names	Application	Brand
GDNF Recombinant Rabbit Monoclonal Antibody [JA93-10]	WB (1:1000)	HUABIO
Phospho-AKT (S473) Recombinant Rabbit Monoclonal Antibody [PSH04-44]	WB (1:2000)	HUABIO
AKT1/2/3 Recombinant Rabbit Monoclonal Antibody [ST48-09]	WB (1:2000)	HUABIO
Bax Recombinant Rabbit Monoclonal Antibody [SZ3-07]	WB (1:1000)	HUABIO
Anti-GFP antibody (ab290)	IF (1:500)	Abcam
Anti-Alpha-synuclein (phospho S129) antibody [EP1536Y]	IF (1:200), WB (1:1000)	Abcam
Anti-Tyrosine Hydroxylase antibody [EP1532Y]	IF (1:200)	Abcam
Donkey Anti-Rabbit IgG H&L (Alexa Fluor® 594)	IF (1:500)	Abcam
Goat anti-rabbit IgG H&L (Alexa Fluor® 488)	IF (1:500)	Abcam
GAPDH (14C10) Rabbit Monoclonal Antibody #2118	WB (1:10000)	Cell Signaling Technology
Anti-rabbit IgG, HRP-linked Antibody #7074	WB (1:10000)	Cell Signaling Technology

WB: Western blot, IF: Immunofluorescence.
